# The Importance of Low Daily Risk for the Prediction of Treatment Events of Individual Dairy Cows with Sensor Systems

**DOI:** 10.3390/s21041389

**Published:** 2021-02-17

**Authors:** Christian Post, Christian Rietz, Wolfgang Büscher, Ute Müller

**Affiliations:** 1 Institute of Animal Science, Physiology Unit, University of Bonn, 53115 Bonn, Germany; ute-mueller@uni-bonn.de; 2 Department of Educational Science, Faculty of Educational and Social Sciences, University of Education Heidelberg, 69120 Heidelberg, Germany; christian.rietz@ph-heidelberg.de; 3 Institute for Agricultural Engineering, Livestock Technology Section, University of Bonn, 53115 Bonn, Germany; buescher@uni-bonn.de

**Keywords:** mastitis, lameness, machine learning, animal welfare

## Abstract

The prediction of health disorders is the goal of many sensor systems in dairy farming. Although mastitis and lameness are the most common health disorders in dairy cows, these diseases or treatments are a rare event related to a single day and cow. A number of studies already developed and evaluated models for classifying cows in need of treatment for mastitis and lameness with machine learning methods, but few have illustrated the effects of the positive predictive value (PPV) on practical application. The objective of this study was to investigate the importance of low-frequency treatments of mastitis or lameness for the applicability of these classification models in practice. Data from three German dairy farms contained animal individual sensor data (milkings, activity, feed intake) and were classified using machine learning models developed in a previous study. Subsequently, different risk criteria (previous treatments, information from milk recording, early lactation) were designed to isolate high-risk groups. Restricting selection to cows with previous mastitis or hoof treatment achieved the highest increase in PPV from 0.07 to 0.20 and 0.15, respectively. However, the known low daily risk of a treatment per cow remains the critical factor that prevents the reduction of daily false-positive alarms to a satisfactory level. Sensor systems should be seen as additional decision-support aid to the farmers’ expert knowledge.

## 1. Introduction

For dairy farms, various sensor systems offer predictions of health data or diseases. These systems offer support for the farmers in their task to identify the animals in need, in the form of veterinary control or treatment. For legal, moral, and ethical reasons, these checks must be carried out daily. In relation to all diseases in dairy cows, most treatments are done for mastitis or lameness [1]. Already a number of studies have examined the classification of cows for mastitis or hoof treatments using single sensors, or combinations of sensors. In many of these studies, the focus is on the first stage of biological validation: the identification of the subjects known to be affected by a given health issue or not, i.e., the Receiver Operator Characteristic (ROC) curve and the indicators sensitivity and specificity [2,3]. Within the framework of these studies, models are often developed using data sets consisting of defined test populations that show a higher proportion of animals with the condition to be predicted (e.g., cows requiring treatment and limited time windows) [4,5].

Fewer studies complete the second stage of validating the developed algorithms on data sets that correspond to a practical situation. In the second stage, the indicators for assessing the predictive quality (called “diagnostic value” in medical test procedures) are determined and used for evaluation. For most procedures, this involves the use of the positive predictive value (PPV), which describes the proportion of false-positives in all positive tests, with the negative predictive value (NPV) used less frequently. The direct relationship between the PPV and the frequency of occurrence of the event to be predicted or classified is seldom highlighted in the studies; the lower the risk of an event being predicted, the lower the PPV or the higher the false-positive rate. In the studies that investigate the possibility of predicting the need for treatment, it is clear that the probability of occurrence of treatments per day per animal is low. Miekley et al. [6] explicitly reported the value related to the practical data was approximately a 0.5% risk for mastitis treatment per animal per day and approximately 7% for lameness treatments, and the authors of Steeneveld et al. [7] found a frequency of 0.04% for mastitis treatments in data from automatic milking systems. The low risk per animal and day for this predictable treatment event leads directly to a low PPV of an alarm list, i.e., a high number of false-positive classifications. In the study of Miekley et al. [6], the values of the PPV for mastitis and lameness treatments were approximately 0.01 and 0.10, and in Steeneveld et al.’s study [7], the resulting PPV was 0.01.

It can be assumed that, by applying the developed algorithms to subgroups in which the event occurs more frequently (also known as risk groups [8]), the PPV is higher and the false-positive rate lower. In human medicine, testing procedures are therefore carried out in these groups or in people with corresponding symptoms in order to increase the probability that the associated event could occur in this group of people. For example, screening tests for chlamydia infections in humans with very high values for sensitivity and specificity of 0.98 and 0.97 (“very high” compared to possible predictive models from livestock farming) in a group of people with a prevalence of 3% could still only achieve a PPV of 0.50, so that half of the tested persons received an incorrect initial diagnosis [9]. For this reason, screening tests, e.g., for chlamydia or HIV, are primarily performed in groups of people with a higher prevalence of the disease under investigation [8,10].

In the context of studies on predictive models of disease indicators in dairy farming, the application of algorithms in risk groups and its effect on the PPV has not yet been investigated. The following risk groups would be conceivable within a herd: cows with a prior treatment in the previous or current lactation [11,12,13], cows with an increased cell count during milk performance testing [14,15], or cows during certain periods of lactation. Data from Koeck et al. [1] specified a higher incidence of clinical mastitis (35% of all cases) in the first 30 days of lactation. In the same study, 22% of all lameness cases occurred in the first 30 days in milk (DIM). For both treatments, the remaining cases were evenly distributed over the rest of the lactation.

However, the application of predictive models to a risk group does not automatically lead to a higher PPV. The authors of Zehner et al. [16] have developed predictive models for calving, i.e., an event in a defined risk period (from seven days before the expected calving date), using data from rumination sensors. Moreover, in these few days before the expected calving, the exact time of calving within the hourly evaluated data (168 h or 24 h before calving) represents a rare event per time unit (0.6% or 4%), so that a high number of false alarms resulted in positive prediction values of only 0.01–0.03 for 168 h or 0.06–0.18 for 24 h [16]. The authors concluded that, despite satisfactory values for sensitivity and specificity, their model was not suitable for practical use because of the low PPV.

The objective of this study was to investigate the importance of the frequency of occurrence of treatments in risk groups and the resulting variation in the PPV for the applicability of classification models in practice. For this, machine-learning models for the classification of mastitis and lameness treatments (i.e., a form of health data available in databases) developed in a previous study [17] were validated using data from other dairy farms.

## 2. Materials and Methods

### 2.1. Data Source

Raw data were collected from three German Holstein dairy farms. The criteria for the selection of these farms were to be able to automatically record milking data (milk yield, milk flow, and conductivity), activity data (pedometer impulse count), as well as feed intake via weighing troughs. This data was transferred in a standardized form to a shared database system, where it was processed and output as CSV files. Per farm, data were used in periods of 3–3.4 years, with average herd sizes of 65–121 cows (see Table 1).

The data collection and processing were carried out analogous to the study in Post et al. [17]. The following types of sensor data were available per cow and day: Milking data (milk yield as the sum of two milkings, milking time, milk flow, conductivity for morning and evening milkings, respectively), feed data (total feed intake, number, average duration of trough visits), pedometer activity (sum of impulses from a pedometer at a 2 h resolution), body weight (kg, averaged for two measurements per day), and animal information (lactation number, days in milk (DIM). Cows with clinical signs were identified during the daily routine and treated by a veterinarian. All treatment data were recorded by farm staff and entered into the database. The milk performance data of the milk recording (milk yield, fat, protein, and lactose content, as well as somatic cell count in 1,000 cells/mL) were recorded monthly for farm A and weekly for farms B and C.

### 2.2. Data Preprocessing

Before further processing, the aggregation, plausibility checks, and adjustment of the data were carried out according to the same scheme of the previous study [17]. Lactations with completely missing values in at least one feature, as well as lactations with less than 28 days in the data, were removed. For farm C, records of treatments were missing for a period of 6 months; data from this period were discarded. Furthermore, on farm C, feed weighing troughs were not installed in all areas of the barn and therefore the records of feed intake of a cow were not continuously available throughout the lactation. Hence only periods with existing data were considered. As in Post et al. [17], additional features were calculated for each variable (except for lactation number, DIM, and monthly milk recording data), which reflected their change over time: Rolling mean (i.e., the moving average) of the last seven days, rolling mean of the previous week, change of the current value to the rolling mean, values of the three previous days, the slope of a linear regression through the last seven days. This step resulted in a total of 182 available features.

**Figure 1:**
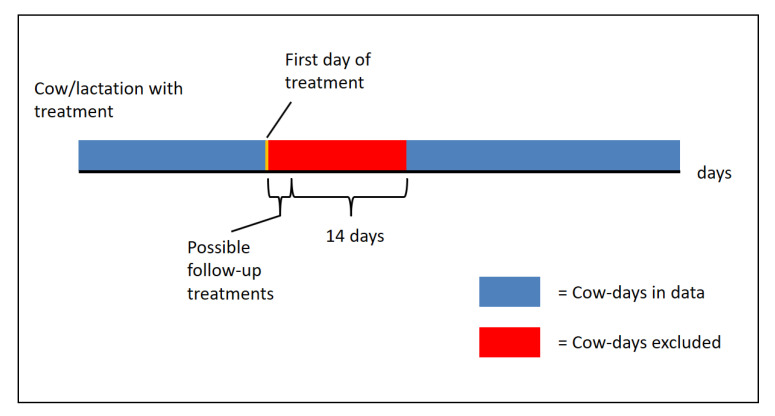
Schematic representation of the extraction of data for cows/lactations with at least one treatment.

Data on treatment records contained information not related to the disease of interest, such as antibiotic treatments for dry-off (udder), hoof care without a diagnosis (claws), estrus synchronization, and silent heat (fertility). These treatment events were ignored. For cows with at least one treatment in a given lactation, the first treatment day was identified for each treatment. A period of 14 days after one treatment, or after the last follow-up treatment if treatment administration occurred over multiple days, was removed from the data, as it was not clear from the data when a cow could be considered “healthy” again, similar to the 14-day exclusion period for follow-up treatments used in the studies by Kamphuis et al. [2] and Jensen et al. [3] (see Figure 1).

Afterward, the day of treatment was moved forward by one day, firstly to exclude the influence of the treatment on the sensor data, and secondly, because, this way, a classification about the cow’s condition tomorrow was already simulated at the end of the previous day.

### 2.3. Training and Testing of the Classification Models

Based on the findings of the previous study [17] four statistical classification models were selected: Random Forest, Logistic Regression, Gaussian Naive Bayes, and ExtraTrees Classifier. These models were part of the Python package Scitkit-Learn [18] and are described in detail in Post et al. [17]. The following steps were performed separately for each of the two categories of mastitis and lameness treatments as the target variable. First, for the training data, all blocks (treatment + previous days) where a treatment other than the target variable was recorded were removed from the data, so that no days were falsely marked as free of treatment. As described in detail in Post et al. [17], the data was normalized (z-score normalization) and feature selection was performed using Sequential Forward Selection (SFS) to identify the most important 20 features per treatment category and facility based on Random Forest mean decrease in impurity.

To evaluate the classification models based on data from the same farm, a 5-fold cross-validation was performed for each farm separately. For this purpose, the cows and lactations were randomly divided into five data sets. For each of these data sets, four were used as training data and the remaining one was used as validation data. The training data set was further reduced to 28 days per lactation, as described in Post et al. [17]. For lactations with a treatment, this was the period before the treatment. For lactations without a treatment, a random period was chosen instead. The models used in this study provided an estimate of the probability that a cow was in need of treatment on a given day. Based on the results from the cross-validation, thresholds for this probability estimate were determined where sensitivity was at least 0.7. These thresholds were stored for later application to the test data. The models were then re-trained on the entire data of one farm and then applied to the data of the two remaining farms. This was done for all cows and days, as well as for only those days on which a cow was classified as a “risk animal” (according to the groups defined in Section 2.4). Alongside this approach, the models were also tested using the combined data from two farms as training data, and then using the remaining farm as testing data, to identify possible differences between these procedures.

### 2.4. Definition of Risk Groups

To increase the frequency of days with treatments and thus improve the classification for mastitis and lameness treatments, the cow-days (i.e., the data from one cow on a particular day) were filtered based on various criteria, further referred to as risk groups (RG). Three different RG limited the testing data to only cow-days with previous treatments:

- RGtreat-SC/PL: Cows with at least one treatment of the same category in the previous lactation
- RGtreat-SC/SL: Cows with at least one previous treatment of the same category in the same lactation
- RGtreat-OC/SL: Cows with at least one previous treatment of another category in the same lactation

**Figure 2:**
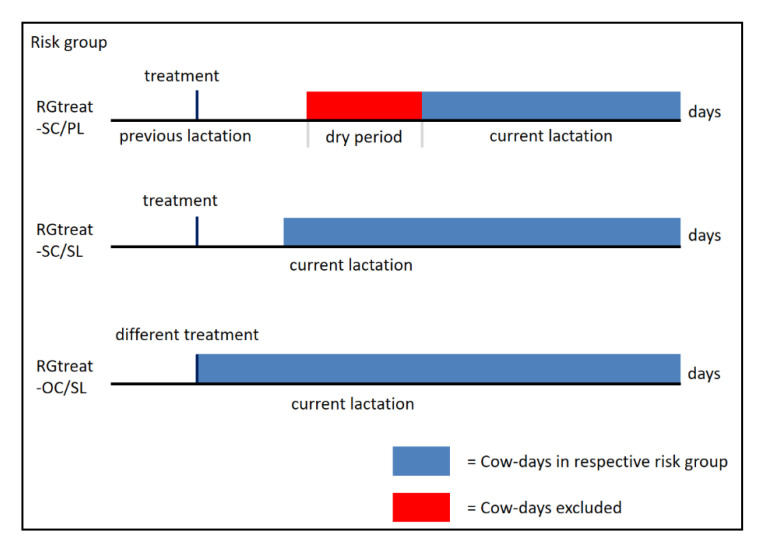
Schematic representation of the assignment of the cow-days to one of the three risk groups: (RGtreat-SC/PL (at least one treatment of the same category in the previous lactation), RGtreat-SC/SL (at least one previous treatment of the same category in the same lactation) and RGtreat-OC/SL (at least one previous treatment of another category in the same lactation).

The classification into a risk group applies to a cow from the occurrence of the respective condition until the end of the current lactation. This scheme is shown in Figure 2. In RGtreat-SC/PL the cow belongs to this group from the day of calving, (DIM 1 in the data). Membership in RGtreat-SC/SL starts as soon as 14 days after the first treatment, and the days from the treatment until that point have been removed from the data.

RG-SCC contained only cow-days where a cow showed an increased somatic cell count (SCC) during the last milk recording (MR). This includes four criteria, which were derived from the udder health indicators of the German Association for Performance and Quality Testing (“Deutscher Verband für Leistungs- und Qualitätsprüfungen e.V.”, DLQ) [19]:

- Cows with a new infection of the udder (defined by SCC > 100,000 with previous SCC of ≤ 100,000)
- Cows with an infection in the first MR after calving (SCC > 100,000), if last SCC in the previous lactation ≤ 100,000
- Heifers with an infection in the first MR after calving (SCC > 100,000)
- Cows with chronic mastitis (three consecutive MR with SCC > 700,000)

**Figure 3:**
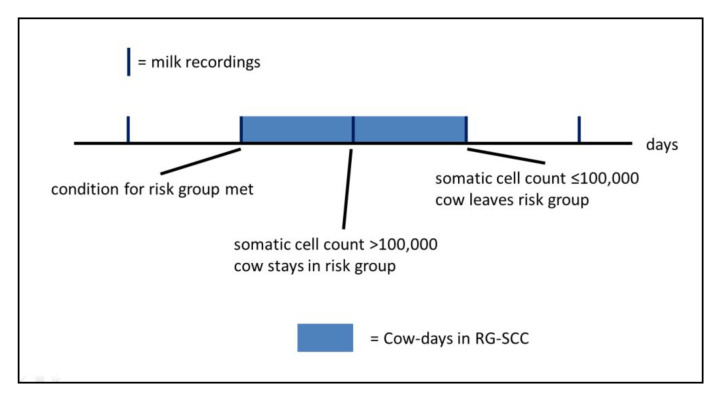
Schematic representation of the assignment of cow-days to the risk group RG-SCC (increased somatic cell count after monthly/weekly milk recording).

The SCC describes the proportion of somatic cells in one mL of milk and provides information about the udder health status of a cow. The critical value for udder health is an SCC of > 100,000 per mL, values above this threshold indicate an infection of the udder [20]. A cow was assigned to RG-SCC from the day of MR if at least one of the above-mentioned criteria was detected. This membership was valid either until an SCC of ≤ 100,000 per mL was detected in a subsequent MR or, if this did not occur, until the end of lactation. This is illustrated in Figure 3.

Lastly, for the formation of the risk group RGtime-100, only cow-days with a value for the DIM of ≤ 100 were included in the test data. In addition, a test data set was also formed using the same procedure, but with the criterion “DIM ≤ 60”, in order to test the effect of even greater limitation.

### 2.5. Evaluation

To assess the classification value of trained models on test data sets in which the event to be classified is available as a reference, the frequency of occurrence of the event to be classified in this test data set is determined. This is done by dividing the number of days with treatments by the total number of all days and then displaying it as a percentage. Secondly, the PPV is calculated to interpret the prediction quality. The level of the PPV depends on the risk that the event, in this case, the treatment, will occur.

**Figure 4:**
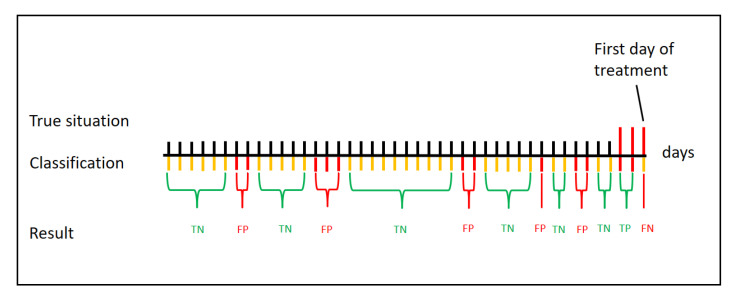
Example classification of days for a single cow with a treatment (last 3 days labeled positive). Yellow bars mean no alert, red bars mean alerts. Green brackets indicate true negative and true positives days, red brackets indicate false-positive and false-negative days.

For each cow and day, the models yielded a probability of belonging to class label “1”, i.e., in need of treatment. These probabilities in combination with the vector of true labels were used to obtain the area under curve (AUC) from the Receiver Operator Characteristic (ROC), as described in detail in Post et al. [17]. In addition to the actual day before a treatment, another two days before treatment were also marked as “treated” in the data, i.e., an alarm was considered true positive within three days before treatment (see Figure 4). Subsequently, the probabilities were compared to the threshold obtained in the model validation to create a vector of binary classifications. This vector was compared with the vector of true labels. Any day with a treatment event could either have an associated alert or not. If an alert was present on a treatment day, it was classified as a true positive (TP), if not, a false-negative (FN); conversely, if an alert was present, that day was a false-positive (FP), and if an alert was not present, the day was a true negative (TN). This is demonstrated in Figure 4.

All values of TP, FP, FN, and TP were summed up for all cows and used for the following calculations:

Sensitivity = TP/(TP + FN)

Specificity = TN/(TN + FN)

Additionally, the block sensitivity was calculated. Here, a cow-day was considered TP if a positive classification was given on at least one of the three days prior to treatment, and FN if none of these three days were detected. This value is always expected to be higher than the sensitivity.

In addition, the positive predictive value (PPV, also referred to as “precision”) was calculated and describes the percentage of correctly classified cows of all cows classified as “treated”.

PPV = TP/(TP + FP)

The results were averaged for all four classification models per treatment category (mastitis or lameness) and per testing farm or per risk group. All results were presented as mean ± 95% confidence interval of the mean, which was calculated with the Python Statsmodels package [21]. Differences between farms and risk groups were performed using Welch’s test due to the violated homogeneity of variance. For multiple comparisons in the post-hoc test, Dunnett-T3 was used. These tests were implemented within SPSS version 26.0 (IBM Corp, Armonk, NY, USA) with significant differences at *p* < 0.05.

## 3. Results

### 3.1. Validation Results

After the preprocessing steps described in Section 2.2, a total of 42,803 cow-days and 48,041 cow-days from 794 individual cows for the mastitis and lameness treatments classification, respectively, remained in the data (see Appendix A Table A3). The 5-fold cross-validation of the classification models for farms A, B, and C on the data from the same farm led to the results shown in Table 2. The Random Forest feature importance obtained during this step is shown in Appendix A Table A1 and Table A2.

The mean AUC was 0.73 for mastitis treatments and was lower for lameness treatments at 0.67. This was consistent with the results from Post et al. [17]. The sensitivity of 0.71 in both treatment categories resulted from the fixed probability threshold of the individual classification models. As expected, the block sensitivities for mastitis were higher at 0.92 for mastitis and 0.85 for lameness.

Table 3 shows the results of the classification (AUC, sensitivity, block sensitivity, and specificity for mastitis and lameness treatments) of all combinations of training and testing farms. The mean AUC for mastitis treatments was higher at 0.72 than for lameness treatments with 0.61. The sensitivities, the level of which was set at 0.7 during validation by fixing the probability threshold values, could not reach this sensitivity value for all testing data sets, but the block sensitivity was on average 0.86 for mastitis treatments and 0.83 for hoof treatments and thus achieved the minimum sensitivity requirement. The mean AUC of 0.72 for mastitis treatments was similar compared to the results from the validation (0.73), whereas for lameness treatments the mean AUC was lower (0.61 compared to 0.67).

The AUCs obtained when using combined training data of two farms, as described at the end of Section 2.3, did not differ significantly from the results of the other approach (mean AUC over all test farms of 0.73 for udder treatments and 0.63 for hoof treatments), so they were not presented separately here.

### 3.2. Positive Predictive Values Depending on the Risk of Occurrence of the Treatments

The mean frequency of cow-days with mastitis and lameness treatments in the data sets was 3.6% and 5.6%, per cow per day, respectively, for all three farms combined (see Table 4). This relationship applied to the present approach resulted in the identification of animals at a higher risk in the following step (for definitions see Section 2.4). Table 4 shows the comparison of the frequency of occurrence for mastitis and lameness treatments in the entire farm data set to the partial data sets of the respective risk groups and risk time.

It is noticeable that the highest increase in the frequency of occurrence or risk for both mastitis and lameness treatments was caused by restricting our analysis to animals that had already undergone a treatment of the same category in the same lactation (RGtreat-SC/SL). Here, the average increase compared to all cows was +9.5 percentage points to 13.5% risk for another mastitis treatment and +7.8 percentage points to 13.2% risk for another lameness treatment. Limitation to cows that had the same category of treatment (mastitis or lameness treatment) in the previous lactation (RGtreat-SC/PL) increased the mean risk for another treatment by 3.6 percentage points and 2.9 percentage points, respectively.

Likewise, a limitation to MR risk factors based on SCC increased the risk for corresponding mastitis treatments on average by 4.6 percentage points (RG-SCC). The grouping RGtreat-OC/SL produced only small increases of 1.6 percentage points and lower. Limiting the risk period to the first 100 DIM (early lactation, RGtime-100) led to inconsistent results. For the mastitis treatments, the risk for corresponding treatment was comparable to that in the total data set (+0.9 percentage points to 4.5%), whereas the risk for lameness treatments was lower in the early lactation of the three experimental farms than over the whole lactation (−0.6 percentage points to 5%). When only the first 60 DIM were considered as the risk period, it did not lead to a change in the frequency of occurrence compared to the limitation of the DIM to ≤ 100 (4.8% frequency of mastitis and 5.2% of claw treatments). The model data for this risk period were, therefore, not listed additionally hereafter.

**Figure 5:**
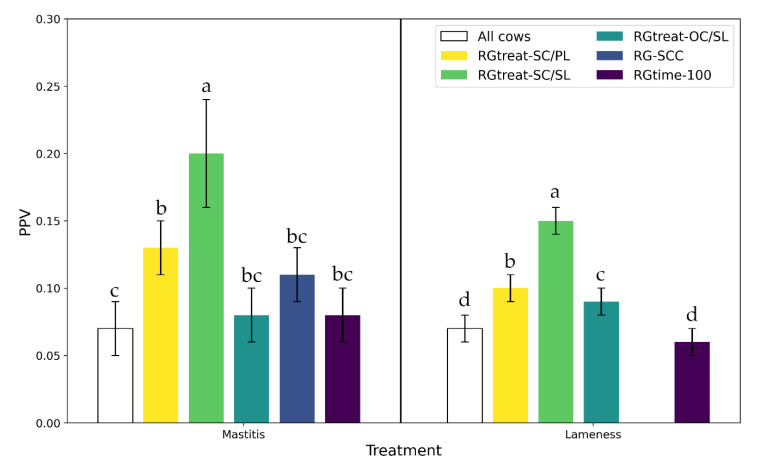
Mean positive predictive value (PPV) from classification results for mastitis and lameness treatments. Error bars indicate the 95%-CI, while letters a, b, c, d indicate significant differences at *p* ≤ 0.05 between treatment categories. RGtreat-SC/PL: at least one treatment of the same category in the previous lactation, RGtreat-SC/SL: at least one previous treatment of the same category in the same lactation, RGtreat-OC/SL: at least one previous treatment of another category in the same lactation, RG-SCC: high SCC in previous milk recording, RGtime-100: DIM ≤ 100.

Table 5 shows the validity criteria AUC, block sensitivity, and specificity per RG in comparison to all cows, while the comparison of the PPV is shown in Figure 5.

It was noticeable that the value of the PPVs reflected the respective frequency of occurrence, i.e., the known low risk for treatment. The highest PPVs were achieved on average in the RGtreat-SC/SL with previous treatments of the same category in the same lactation; for mastitis treatments on average a PPV of 0.20 and for lameness treatments of 0.15. All other PPVs were significantly lower. However, it was noticeable that for AUC both in the classification of mastitis treatments (0.65) and lameness treatments (0.55), significantly lower values were obtained only in RGtreat-SC/SL compared to the test data of all cows. The sensitivity, block sensitivity, and specificity did not differ between the risk groups within the treatment categories; therefore the sensitivity is not shown in Table 5. In the following paragraphs, the respective risk groups whose AUCs and PPVs showed statistically significant differences are described individually.

#### 3.2.1. Predictive Value of the Models for the Respective Treatment Risk Groups (RGtreat)

Due to the higher frequencies of occurrence in the narrowed data to the risk group of animals already treated for mastitis or lameness in the past, the PPVs increased from 0.07 to 0.13 for mastitis treatments and from 0.07 to 0.10 for lameness treatments. The AUC of the models for RGtreat-SC/PL were comparable to the results for all cows and were 0.69 for udder and 0.59 for lameness treatments.

When applying the trained models to RGtreat-SC/SL (cows with at least one previous treatment of the same category in the same lactation), the higher PPVs of 0.20 for mastitis treatments and 0.15 for lameness treatments, compared to all other risk groups, stood out due to the highest frequency of days with treatment compared to the other risk groups. The AUC for predicting mastitis treatments of RGtreat-SC/SL was 0.65 and for lameness treatments 0.55, significantly lower than the results for the respective treatments in the data of all cow-days. At the same time, RGtreat-SC/SL was the group with the lowest number of cow-days in the test data (4251 days for the mastitis treatments and 8417 days for the lameness treatments, see Table 5). The combinations of block-sensitivities and specificities were in similar ranges as in RGtreat-SC/PL., i.e., animals with corresponding treatments in past lactation.

For RGtreat-OC/SL, the trained models were applied to the risk group of cows that had at least one treatment of another category (i.e., not also a treatment for mastitis, lameness, or metabolic disorders) in the same lactation. For both mastitis and lameness treatments, the AUC of 0.69 and 0.59 did not differ significantly from the AUC of the classification for all cow-days. As already shown in Table 4, this restriction did not lead to a significant increase in the frequency of days with treatment (+0.8% for mastitis treatments and +1.1% for lameness treatments), so the PPVs were in the range of 0.09 for mastitis treatments and 0.09 for lameness treatments.

#### 3.2.2. Predictive Value of the Models for the Risk Group According to the Information on SCC from Milk Recording (RG-SCC)

The SCC categories explained in Section 2.4 allowed the classification of the animals into the risk group RG-SCC. The mean values for AUC (0.71), block sensitivity, and specificity were comparable to those of the validation results. However, the mean PPV of 0.11 did not differ significantly from the PPV for all cows.

#### 3.2.3. Predictive Value of Models in Early Lactation as Risk Time Period (RGtime)

However, as shown in Table 4, the frequency of mastitis treatments in the risk period of early lactation (≤ 100 DIM) in all three farms was comparable to the treatment frequency over the entire lactations. In terms of treatments for lameness, the risk of treatment was lower in the early lactation period than over the entire lactation period. Accordingly, the AUC values (0.73 for mastitis treatments and 0.58 for lameness treatments) and the PPV (0.08 for mastitis treatments and 0.06 for lameness treatments) showed no significant changes compared to the evaluation without time limitation. Reducing the risk period to only 60 DIM resulted in AUC values of 0.74 ± 0.03 for mastitis treatments and 0.60 ± 0.02 for lameness treatments, and a PPV of 0.07 ± 0.01 for mastitis treatments and 0.08 ± 0.01 for lameness treatments. Again, these values did not differ significantly.

## 4. Discussion

### 4.1. Validation Results

The aim of the validation was to test the applicability of the models developed in Post et al. [17] for the classification of mastitis and hoof treatments on data from two other experimental farms to simulate the usage of trained models on other unknown datasets of additional farms.

The AUCs of 0.73 for mastitis treatments and 0.67 for lameness treatments obtained by cross-validation are comparable to values obtained in studies that have performed classifications with comparable sensors. For the detection of mastitis, a study with automatic milking system (AMS) data and additional cow information (parity, DIM, season, SCC history, and clinical mastitis history) over two years achieved a range of AUC values between 0.62 and 0.78 [7]. For the detection of lameness treatments, AUC values between 0.66 and 0.75 were achieved in a study by Kamphuis et al. [2] using data from a total of 4904 cows (from five farms) and the features live weight, activity and milk yield, and milking duration. In a further study on the detection of lameness treatments, an AUC of only 0.60 was obtained from a data set of 315 cows for a comparable time window of three days prior to each treatment [22]. It should be noted that an AUC of 0.70 and above describes a “strong model”, while a value of 0.60 and below describes only a “weak model” [23].

On average, higher AUC values are obtained for the classification of mastitis diseases or treatments than for the prediction of lameness treatments. This can be explained by the high importance of the feature “SCC of the last milk recording (MR)”. As already discussed in [19], the feature “SCC” is directly related to the event “mastitis”. This could be confirmed on all three test farms. When comparing the individual models, it was noticeable that farms B and C showed a higher relative importance of the cell count with 0.22 compared to farm A with 0.13. This can be explained by the higher frequency of MR (B and C: weekly vs. A: monthly). The periods between the measured cell count and the mastitis treatments were shorter due to the weekly MR and the correlation was, therefore, more direct, whereas the monthly MR increases the probability of an intermediate healing or new infection, which is then not found in the data. For the same reason, the AUC values of the mastitis models were not significantly lower when the trained models were tested from one farm to the other farms (from 0.73 on the training farm to an average of 0.72 on the test farms). As shown in Table 3, the lower frequency of MR on farm A had no negative influence on the AUC when used for training the models, compared to farms B and C. In comparison, the trained models for classifying lameness treatments with AUC values averaging 0.67 are considered “weak models” and, therefore, their practical usefulness is questionable. This is further reinforced by the application of the trained models to other farms or their data, as the AUC values are significantly reduced to an average of 0.61. Since no available features in the detection of lameness treatments are directly or specifically related to the event, the operational differences are much more relevant for the model quality.

### 4.2. Positive Predictive Values Depending on the Probability of Occurrence of the Treatments

A common feature of the mentioned studies [6,7,22] is the high proportion of false-positive alarms (also referred to as error rate). This value was 0.99 for mastitis and 0.89 for lameness treatments in the results from Miekley et al. [22]. The study of Steeneveld et al. [7] included 52 true positive and 3636 false-positive alarms, which led to an error rate of approximately 0.99. The PPV in our own study of 0.07, which corresponds to a 0.93 error rate, is due to the low frequency of days with treatment in the test data, which was 3.5% and 5.4% for mastitis and lameness treatments, respectively. In other studies, the ratio of treated to non-treated cows was artificially increased, i.e., the data were sampled, e.g., by pairing cows [5], by excluding unclear cases of lameness [24,25], or by considering shorter periods before treatment [2,4]. From the perspective of the developers of these models, these measures are justified because sensitivity and specificity are hardly influenced by the frequency of occurrence of the target trait. However, these results do not reflect the situation of the application on a practical farm. Furthermore, Post et al. [17] could show that even different up- and downsampling methods for balancing training data during application to unknown, realistic data had no influence. Thus, it becomes clear that, despite sufficient model quality, the frequency of occurrence of the event to be predicted substantially influences the magnitude of the predictive values and thus the share of animals reported as positive. As known from medicine and other fields [8,9], the application of test procedures and, accordingly, algorithms in groups where the risk for the event to be predicted is higher, allows the ratio between correct and false-positive reports to improve, i.e., the PPV becomes higher. In the risk groups analyzed, the question arises whether this improves the ratio in such a way that an implementation of this approach can be recommended.

#### 4.2.1. Classification of Cows with a Previous Treatment (RGtreat)

Our own results have shown that cows with mastitis or lameness treatment have a higher chance of needing to be treated again in the next lactation (RGtreat-SC/PL) or at a later stage of lactation (RGtreat-SC/SL). In other studies, an increased risk of further mastitis was found in cows that had already been infected with the cow-associated pathogen *Staphylococcus aureus* [26], as well as in cows with a past infection with the environmentally associated pathogens *Streptococcus uberis* [26] and *Escherichia coli* [27], in whose study approx. 13% of all *E. coli* infections already had an infection in the same udder quarter. In [11], the odds ratio of mastitis was found to be up to 5.9 if at least one previous treatment was given in the current lactation. By narrowing the data to cows with a previous treatment in the same lactation, the frequency of occurrence in our own study was increased to 13.2% from 3.5% of days with treatment. This means a 3.7-fold higher risk of mastitis for this group. Another study found an odds ratio of 4.15 for mastitis incidence in the first 120 days of lactation for previous clinical mastitis [12]. These results suggest that cows or udder quarters are more likely to develop mastitis again [26,27]. However, in another study by Hammer et al. [28], no statistical correlation between the risk of mastitis and previous treatments that were more than 30 days old was found in 245 cases of mastitis.

An increased risk for subsequent treatments in the following lactation was also found by other authors. In a study of 402 cows, cows treated in the previous lactation were found to be 1.7 times more likely to develop subclinical mastitis in the first 60 days in the next lactation [29]. When restricted to animals treated in the last 60 days of the previous lactation, the risk there increased 4.9-fold. Another study with data from 350 Norwegian dairy herds and a total of 6046 cows in their second lactation [14] showed an increased risk (1.5-fold) when mastitis treatment was given in the first lactation. Limiting the risk to animals with mastitis treatment in the previous lactation (RGtreat-SC/PL) achieved a 2-fold increase in risk to 7.1% in our own study. This shows that cows with mastitis treatment also carry a higher risk into the next lactation due to individual susceptibility to pathogens or the persistence of a subclinical infection over the dry period [11]. However, this risk is reduced to some extent by the possibility of udder healing in the dry period through appropriate therapies [30], compared to the follow-up treatments within one lactation.

The risk that a cow will need to be treated again was also elevated for lameness treatments for both RGtreat-SC/PL and RGtreat-SC/SL. In another study with 600 cows over 44 months, a high range of positive odds ratios between 2.5 and 23 for all types of lameness diagnoses was found for the probability of a cow needing re-treatment [13]. A different study of over 7600 cows from 23 dairy farms found significant positive effects of prior lameness treatment on both at dry-off (2.5 times higher risk) and next lactation (twice the risk) for claw horn disruption lesions [31]. In other studies, this association has also been established for treatments for sole ulcers, white line defects, and digital dermatitis [13,32]. This is the case when treatment of the clinical symptoms does not address the underlying cause sufficiently, e.g., a thinned digital cushion [13].

The AUCs of RGtreat-SC/PL and RGtreat-OC/SL did not differ significantly from those models applied to all cows. Only the AUCs after application in RGtreat-SC/SL showed significantly lower values in both treatment categories. This was due to the combination of low numbers of cow-days in the corresponding test data (see Appendix A Table A3) and the restriction of the test data to a subgroup with a different distribution of features for days with and without treatment than in the whole test data. This introduces a sampling bias into the classification, which has a negative effect on AUC, especially in small data sets [33,34]. At the same time, in this RGtreat-SC/SL, the risk of repeated treatment for mastitis or lameness was highest. Accordingly, PPVs in this RGtreat-SC/SL had the significantly highest values compared to the other groups. This means that they have the greatest potential for reducing false-positives compared to the other RGs, yet the PPVs were not in a range satisfactory for practical use, with 0.20 for mastitis and 0.15 for lameness treatments.

RGtreat-OC/SL narrowed the data down to cows that had already undergone a different treatment in the same lactation. A study on genetic correlations found a comparatively low correlation of 0.32 ± 0.07 between the occurrence of mastitis between lactation days −10 to 50 and other treatments (fertility disorders, metabolic diseases, and lameness) in the period up to 100 DIM [35]. Another study by Hossein-Zadeh and Ardalan [12] found odds ratios for clinical mastitis in the first 120 DIM of 57,300 Holstein cows, 9.45 with previous retained placenta and 12.36 with previous milk fever. The association between the retained placenta and later clinical mastitis has before been quantified by [36] with a 1.5-fold higher risk for mild and 5.4-fold higher risk for severe mastitis, respectively. Acidosis can act as a trigger for laminitis, which then develops into lameness [37]. A study by Berge and Vertenten [38] with 131 Dutch farms found odds ratios at previous ketosis of 1.9 for mastitis treatments and 1.7 for lameness treatments in the rest of the lactation. The authors of [39] found a significant doubling of the frequency of interdigital dermatitis in cows with previous endometritis in the same lactation based on data with 2109 lactations, but the data showed no correlation between other previous diseases and mastitis. Our own results for RGtreat-OC/SL could only cause a small increase in the frequency of occurrence of mastitis and lameness treatments, and consequently no higher PPVs by limiting the animals to those treated against diseases from other disease categories (with otherwise comparable AUC values). Since the cows remained in this risk group for the remainder of the lactation, the effects of these pre-treatments are too small in relation to the total data at the daily level.

#### 4.2.2. Classification of Cows with Increased SCC After Milk Recording (RG-SCC)

Several studies have investigated the association between increased SCC in MR and the subsequent occurrence of mastitis. In Whist and Østerås [14], a 1.9-fold higher risk of clinical mastitis was found for SCC > 200,000 cells/mL in the first MR after calving. The authors also found a 1.7-fold higher risk of developing mastitis in the second lactation with a geometric mean between 400,000 and 800,000 of the last three MR cell counts before the second calving [14]. In a study by Steeneveld et al. [15], the relationship between the previous month’s SCC and the geometric mean of all MR test days of the previous lactation with mastitis treatments was examined using data from almost 40,000 cows and 8500 mastitis cases. The significant odds ratios here were 1.33 and 1.15 for elevated SCC (> 200,000) in the preceding MR and previous lactation on average, respectively, which signaled a slightly increased risk of a subsequent mastitis treatment.

RG-SCC showed a comparable AUC as an indicator of model quality, but to a significantly lower PPV compared to RGtreat-SC/SL. The reason for this is that, whereas clinical symptoms were present at one time during pre-treatment, a SCC of > 100,000 and thus a risk after MR is not necessarily associated with clinical symptoms, and therefore no treatment is performed. Thus, the limitation to this risk group and the application of the classification algorithms would not lead to any added value other than the animal listings themselves, which are conspicuous in the context of MR with regard to udder health.

#### 4.2.3. Classification of Cows in Early Lactation (RGtime-100)

Only cow-days within the first 100 DIM were classified as this last risk group. It is known that treatments for mastitis are more common in early lactation [12,40]. The study by Hammer et al. [28] found in 245 cows that the odds ratio in cows over 100 DIM dropped to only 0.3 compared to the reference group between 10 and 20 DIM. However, this odds ratio was also only 0.4 between 30 and 100 DIM. The odds ratio for clinical mastitis decreased after the first month of lactation [15], but after the first three months (after about 100 DIM) the odds ratio was still 1.9 for primiparous and 3.6 for multiparous cows, compared to lactation month 8 and higher as reference. In terms of lameness treatments, in a study of 2100 cows over three years, these were most common between 61 and 150 DIM and least common between 16 and 60 DIM [41]. However, these data are from only one farm, so a farm effect cannot be excluded.

In our own study, the restriction only to animals in the first 100 DIM did not lead to an increase in the frequency of occurrence and thus no effect on the PPV. The effects in the quoted studies often reported shorter time windows after calving with higher risk. This was also investigated in our own study (60 DIM) but did not lead to any change in the frequency of treatments. In line with the findings from the other risk groups, this narrowing of the data set also did not lead to any improvement in predictive values or false alarms.

### 4.3. General Discussion

The results of our own study have shown that a limitation to risk groups can improve the PPVs of a daily detection of individual animals in need of treatment using sensor data up to 0.20 PPV (i.e., 80% error rate). However, the suitability for satisfactory practical use remains questionable. In contrast to the minimum requirements for the model quality criteria of the models that can be used in practice (i.e., a sensitivity of 0.70–0.80 and a specificity of 0.99 [42]), there are no recommendations for a minimum PPV to be achieved. A recent survey of practicing farmers’ preferences for the performance of a lameness detection system included options for the percentage of false alarms from 0% to 15%, corresponding to a PPV of 0.85–1.00 [43]. Although not explicitly asked for tolerable, but rather for preferred values, this shows the discrepancy between user expectations and the actual percentage of false alarms. The study by Steeneveld et al. [7] on the reduction of false-positive mastitis alarms of an AMS showed that despite a very good test characteristic of their model of 0.70 sensitivity and 0.98 specificity and a reduction of false-positive alarms by up to 35 percentage points, the PPV was still only 0.03 due to the low frequency of cow days with mastitis in the data (227 out of 508,517). This shows that despite all optimization attempts, the known low risk of one treatment (i.e., a form of health data available in databases) per animal per day remains the main factor influencing the prediction. As already discussed in Van De Gucht et al. [42] and Zehner et al. [16], it is not possible to use classification models of sensor systems as the only tool to find the animals that need treatment or need special care.

## 5. Conclusions

The objectives of this study were to demonstrate the importance of applying classification models for cows in need of treatment to practical data sets and to highlight the importance of the low frequency of occurrence of the trait “treatment” in relation to individual cows and days, based on the assignment of cows to risk groups.

Within those risk groups, the frequency of occurrence of the target variable “treatment” and the respective PPV increased accordingly. This influence was the largest when applying the algorithms to the risk group of cows with previous treatment in the same lactation, but even the highest achieved PPV of 0.20 is not sufficient for the prediction of mastitis and lameness treatments in practice. The critical factor influencing the prediction remains, despite all optimization variations with respect to model validity, the known low risk of a treatment per animal per day. It can be assumed that this also applies to other health or disease data. This requires rethinking and specific information about the fact that the detection of cows in need of treatment within a herd is not possible through sensor data and the corresponding algorithms only, but requires additional expert knowledge. However, if the responsible person already has certain animals in focus during the day, the existing animal-specific (sensor) data can be important decisional support.

## Figures and Tables

**Table 1:** Overview of the three farms and the amount of data used.

Farm	Time Period	Raw Data Size ^1^	Mean Herd size	Mean Daily Milk Yield (kg)	Mean Lactation Number ^2^
A	June 1, 2015–October 20, 2018	80,307	65	31.3	2.4 ± 1.7
B	January 1, 2014–May 31, 2017	203,421	163	36.6	2.4 ± 1.4
C	January 1, 2017–December 31, 2019	133,270	121	35.4	2.3 ± 1.6

^1^ records per cow and day, ^2^ ± standard deviation.

**Table 2:** Mean area under curve (AUC), sensitivity, block sensitivity, and specificity (± 95%-CI) for 5-fold-cross validation data. Training and testing were performed on the same farm.

Treatment	Farm	AUC ^1^	Sen. ^2^	Block Sen. ^3^	Spe. ^4^
Mastitis	A	0.70 ± 0.08 ^b^	0.72 ± < 0.01 ^a^	0.93 ± 0.02 ^ab^	0.59 ± 0.13 ^b^
	B	0.80 ± 0.02 ^a^	0.71 ± < 0.01 ^b^	0.89 ± 0.02 ^b^	0.74 ± 0.04 ^a^
	C	0.70 ± 0.04 ^b^	0.72 ± < 0.01 ^a^	0.94 ± 0.03 ^a^	0.59 ± 0.08 ^b^
	Mean	0.73 ± 0.04	0.71 ± < 0.01	0.92 ± 0.02	0.64 ± 0.06
Lameness	A	0.72 ± 0.02 ^a^	0.71 ± < 0.01 ^c^	0.85 ± 0.03	0.59 ± 0.03 ^a^
	B	0.66 ± 0.02 ^b^	0.70 ± < 0.01 ^b^	0.83 ± 0.03	0.51 ± 0.04 ^ab^
	C	0.63 ± 0.07 ^b^	0.72 ± < 0.01 ^a^	0.86 ± 0.03	0.48 ± 0.09 ^b^
	Mean	0.67 ± 0.03	0.71 ± < 0.01	0.85 ± 0.01	0.53 ± 0.04

^1^ area under ROC-curve; ^2^ sensitivity; ^3^ block sensitivity; ^4^ specificity; ^a,b,c^ superscript letters indicate significant differences at *p* ≤ 0.05 between farms within treatment categories.

**Table 3:** Mean area under curve (AUC), sensitivity, block sensitivity, and specificity (± 95%-CI) for the classification of mastitis and lameness treatments of all cows. Models were trained on one farm and then tested on the two other respective farms.

	Farm					
Treatment	Training	Test	AUC ^1^	Sen. ^2^	Block Sen. ^3^	Spe. ^4^
Mastitis	A	B	0.78 ± 0.03 ^a^	0.60 ± 0.19 ^ab^	0.82 ± 0.13 ^ab^	0.80 ± 0.23 ^a^
		C	0.70 ± 0.05 ^abc^	0.47 ± 0.18 ^b^	0.72 ± 0.17 ^ab^	0.80 ± 0.23 ^a^
	B	A	0.73 ± 0.01 ^b^	0.62 ± 0.14 ^b^	0.86 ± 0.12 ^ab^	0.72 ± 0.11 ^ab^
		C	0.69 ± 0.02 ^c^	0.55 ± 0.07 ^b^	0.80 ± 0.08 ^b^	0.71 ± 0.11 ^ab^
	C	A	0.71 ± 0.06 ^abc^	0.91 ± 0.07 ^a^	1.00 ± < 0.01 ^a^	0.27 ± 0.20 ^bc^
		B	0.74 ± 0.02 ^ab^	0.89 ± 0.13 ^a^	0.97 ± 0.04 ^a^	0.30 ± 0.26 ^b^
	Mean		0.72 ± 0.02	0.67 ± 0.08	0.86 ± 0.05	0.60 ± 0.11
Lameness	A	B	0.62 ± 0.02 ^bc^	0.81 ± 0.12 ^a^	0.90 ± 0.08 ^ab^	0.28 ± 0.16 ^bc^
		C	0.60 ± 0.02 ^c^	0.84 ± 0.11 ^a^	0.89 ± 0.08 ^ab^	0.26 ± 0.13 ^bc^
	B	A	0.66 ± 0.02 ^ab^	0.74 ± 0.05 ^a^	0.87 ± 0.05 ^a^	0.45 ± 0.08 ^abc^
		C	0.56 ± 0.02 ^d^	0.64 ± 0.02 ^ab^	0.76 ± 0.03 ^b^	0.43 ± 0.06 ^abc^
	C	A	0.66 ± 0.02 ^a^	0.66 ± 0.11 ^ab^	0.83 ± 0.13 ^ab^	0.56 ± 0.13 ^ab^
		B	0.57 ± 0.04 ^d^	0.53 ± 0.09 ^b^	0.73 ± 0.13 ^ab^	0.58 ± 0.15 ^a^
	Mean		0.61 ± 0.02	0.71 ± 0.05	0.83 ± 0.04	0.42 ± 0.06

^1^ area under ROC-curve; ^2^ sensitivity; ^3^ block sensitivity, ^4^ specificity; ^a,b,c^ superscript letters indicate significant differences at *p* ≤ 0.05 between farm combinations within treatment categories.

**Table 4:** Frequency (%) of cow-days with a mastitis treatment and lameness treatment in the testing data for all cows (no specific risk group).

Treatment	Farm	All Cows	RGtreat-SC/PL	RGtreat-SC/SL	RGtreat-OC/SL	RG-SCC	RGtime-100
Mastitis	A	2.5	5.4	12.0	3.0	5.4	2.9
	B	4.8	8.1	13.6	6.5	9.6	4.9
	C	3.8	8.3	14.0	5.1	5.1	5.0
	All	3.6	7.8	13.5	5.7	8.2	4.5
Lameness	A	4.3	7.0	12.9	6.0	6.8	3.1
	B	5.5	8.5	13.2	6.6	6.4	5.2
	C	6.8	9.9	13.3	7.9	7.4	6.1
	All	5.6	8.5	13.2	6.6	6.7	5.0

RGtreat-SC/PL: at least one treatment of the same category in the previous lactation, RGtreat-SC/SL: at least one previous treatment of the same category in the same lactation, RGtreat-OC/SL: at least one previous treatment of another category in the same lactation, RG-SCC: high SCC in previous milk recording, RGtime-100: DIM ≤ 100.

**Table 5:** Comparison of classification results for mastitis and lameness treatments (mean area under the curve (AUC), block sensitivity, and specificity ± 95%-CI), as well as the total number of cow-days in the data and the treatment frequency in % in the testing data for all cows, as well as the risk groups.

Risk Group	Cow-days Total	Frequency of Treatment Days (%)	AUC ^1^	Block Sen. ^2^	Spe. ^3^
Mastitis					
All cows	42,803	4.1	0.72 ± 0.02 ^a^	0.86 ± 0.05	0.60 ± 0.11
RGtreat-SC/PL	6,633	7.8	0.69 ± 0.02 ^a^	0.83 ± 0.06	0.57 ± 0.11
RGtreat-SC/SL	4,251	13.5	0.65 ± 0.02 ^b^	0.81 ± 0.06	0.57 ± 0.11
RGtreat-OC/SL	17,802	5.7	0.69 ± 0.02 ^ab^	0.87 ± 0.03	0.63 ± 0.06
RG-SCC	10,414	8.2	0.71 ± 0.02 ^a^	0.86 ± 0.05	0.58 ± 0.11
RGtime-100	18,289	4.5	0.73 ± 0.02 ^a^	0.88 ± 0.06	0.60 ± 0.11
Lameness					
All cows	48,041	5.6	0.61 ± 0.02 ^a^	0.83 ± 0.04	0.42 ± 0.06
RGtreat-SC/PL	9,587	8.5	0.59 ± 0.02 ^a^	0.83 ± 0.04	0.43 ± 0.06
RGtreat-SC/SL	8,417	13.2	0.55 ± 0.02 ^b^	0.79 ± 0.04	0.40 ± 0.06
RGtreat-OC/SL	18,137	6.6	0.59 ± 0.02 ^a^	0.72 ± 0.05	0.55 ± 0.04
RGtime-100	20,044	5.0	0.58 ± 0.01 ^ab^	0.80 ± 0.04	0.42 ± 0.06

^1^ Area Under ROC-Curve; ^2^ Block Sensitivity; ^3^ Specificity; ^a,b^ superscript letters indicate significant differences at *p* ≤ 0.05 between farm combinations within treatment categories. RGtreat-SC/PL: at least one treatment of the same category in the previous lactation, RGtreat-SC/SL: at least one previous treatment of the same category in the same lactation, RGtreat-OC/SL: at least one previous treatment of another category in the same lactation, RG-SCC: high SCC in previous milk recording, RGtime-100: DIM ≤ 100.

## Data Availability

The data used in this study are not publicly available due to a confidentiality agreement.

## Appendix A

**Table A1:** Random Forest feature importance for mastitis treatments (Mean ± 95% CI), obtained during 5-fold cross validation.

Farm					
A		B		C	
Feature	Importance	Feature	Importance	Feature	Importance
Last milk recording SCC	0.13 ± 0.07	Last milk recording SCC	0.22 ± 0.03	Last milk recording SCC	0.22 ± 0.04
Highest milk flow p.m., slope	0.11 ± 0.04	Milk yield, RMdiff	0.14 ± 0.02	Conductivity a.m., RMdiff	0.14 ± 0.03
Conductivity p.m., slope	0.10 ± 0.04	Milk yield p.m., RMdiff	0.09 ± 0.01	Conductivity p.m., RMdiff	0.07 ± 0.02
Milking duration a.m.	0.06 ± 0.02	Milk yield, slope	0.08 ± 0.02	Day/night ratio activity, RM	0.05 ± 0.01
Conductivity p.m., RMdiff	0.06 ± 0.02	Milk yield p.m., slope	0.07 ± 0.01	Feed intake, RMdiff	0.05 ± 0.01
Milk yield, RMdiff	0.06 ± 0.03	Feeding time with intake	0.06 ± 0.01	Milk yield, RMdiff	0.05 ± 0.01
Feed intake, RMdiff	0.05 ± 0.01	Milk yield a.m., RMdiff	0.06 ± 0.01	Last milk recording lactose	0.05 ± 0.01
Feed intake, RMprev	0.05 ± 0.02	Conductivity p.m., RMdiff	0.05 ± 0.01	Feed intake, slope	0.04 ± 0.01
DIM	0.04 ± 0.01	Conductivity p.m., slope	0.04 ± 0.01	Conductivity p.m., slope	0.04 ± 0.01
Feed intake, slope	0.04 ± 0.01	Conductivity a.m., RMdiff	0.03 ± 0.00	Activity (Min), slope	0.04 ± 0.01
Milking duration a.m., diff	0.04 ± 0.02	Body weight, slope	0.02 ± 0.01	Feeding time with intake, slope	0.03 ± 0.01
Milk yield	0.04 ± 0.01	Feeding time with intake, slope	0.02 ± 0.01	Body weight, RMdiff	0.03 ± 0.00
Body weight	0.04 ± 0.01	Milking duration p.m., RMdiff	0.02 ± 0.00	Feed intake, d-1	0.03 ± 0.00
Conductivity a.m., RMdiff	0.04 ± 0.01	Highest milk flow a.m.	0.02 ± 0.00	Day/night ratio activity, RMprev	0.03 ± 0.01
Day/night ratio activity, RM	0.03 ± 0.01	Day/night ratio activity, RMprev	0.02 ± 0.01	Milk flow p.m., RMdiff	0.03 ± 0.01
Highest milk flow a.m.	0.02 ± 0.01	Milk yield	0.02 ± 0.01	Milking duration a.m., slope	0.03 ± 0.01
Highest milk flow a.m., RMdiff	0.02 ± 0.01	Feeding visit duration, RMprev	0.01 ± 0.01	Feeding visit duration, RMprev	0.02 ± 0.01
Activity (max), RMprev	0.02 ± 0.01	Day/night ratio activity, RM	0.01 ± 0.00	Activity Sum, RMprev	0.02 ± 0.01
Activity (Sum)	0.02 ± 0.01	Body weight, RMdiff	0.01 ± 0.00	Activity (Max), RM	0.02 ± 0.00
Activity (Sum) p.m., RMprev	0.02 ± 0.01	Feed intake, RMprev	0.01 ± 0.00	Activity (Sum) p.m., RM	0.01 ± 0.01

For variable descriptions, see [17].

**Table A2:** Random Forest feature importance for lameness treatments (Mean ± 95% CI), obtained during 5-fold cross validation.

Farm					
A		B		C	
Feature	Importance	Feature	Importance	Feature	Importance
Feeding time with intake	0.13 ± 0.02	Feeding time with intake	0.18 ± 0.01	Feeding time with intake	0.11 ± 0.02
Feeding time with intake, slope	0.11 ± 0.03	Feed intake	0.12 ± 0.01	Feeding time with intake, slope	0.09 ± 0.03
Feeding visits	0.08 ± 0.01	Feeding time with intake, slope	0.11 ± 0.02	Feed intake, slope	0.07 ± 0.02
Body weight, slope	0.08 ± 0.02	Feeding visit duration, RMprev	0.06 ± 0.01	Last milk recording SCC	0.07 ± 0.04
Body weight, RMdiff	0.08 ± 0.03	Feed intake, slope	0.06 ± 0.02	Milk yield	0.07 ± 0.01
Feeding visit duration, RMdiff	0.07 ± 0.02	Last milk recording fat	0.06 ± 0.01	Feed intake, d-1	0.07 ± 0.02
Feed intake, slope	0.06 ± 0.00	Feed intake per visit, RMprev	0.04 ± 0.01	Body weight, slope	0.06 ± 0.02
Activity (Min), RM	0.06 ± 0.03	Feed intake, RMprev	0.04 ± 0.01	Highest milk flow a.m., RMdiff	0.04 ± 0.02
Feeding visit duration, slope	0.05 ± 0.02	Milk yield	0.04 ± 0.02	DIM	0.04 ± 0.01
Feeding visit duration, d-1	0.04 ± 0.02	Activity, slope	0.04 ± 0.01	Body weight	0.04 ± 0.01
Last milk recording lactose	0.04 ± 0.01	DIM	0.03 ± 0.01	Activity (Sum) p.m., RM	0.04 ± 0.01
Acitivity, 3 highest (Sum), slope	0.03 ± 0.02	Feed intake per visit, slope	0.03 ± 0.01	Conductivity a.m., RMdiff	0.04 ± 0.01
Feed intake, RMprev	0.03 ± 0.01	Last milk recording SCC	0.03 ± 0.01	Feeding visits, slope	0.04 ± 0.01
Activity (Max), RMprev	0.03 ± 0.01	Activity (Max), RMprev	0.03 ± 0.01	Activity (Min), RMprev	0.04 ± 0.01
DIM	0.02 ± 0.01	Day/night ratio activity, RMprev	0.03 ± 0.01	Feeding visit duration, RMprev	0.04 ± 0.01
Milk yield	0.02 ± 0.00	Day/night ratio activity, RM	0.02 ± 0.01	Day/night ratio activity, RMprev	0.03 ± 0.01
Milking duration a.m.	0.02 ± 0.01	Feeding visit duration, RMprev	0.02 ± 0.01	Day/night ratio activity, RM	0.03 ± 0.01
Conductivity p.m., slope	0.02 ± 0.01	Body weight, slope	0.02 ± 0.01	Last milk recording fat	0.03 ± 0.01
Day/night ratio activity, RM	0.01 ± 0.01	Activity (Min), slope	0.02 ± 0.00	Activity (Max), slope	0.03 ± 0.01
Day/night ratio activity, RMprev	0.01 ± 0.01	Feeding visit duration, slope	0.02 ± 0.01	Milking duration a.m., slope	0.02 ± 0.00

For variable descriptions, see [17].

**Table A3:** The number of cow-days in the test data for all cows, as well as in the respective risk groups.

			Risk Group				
Treatment	Farm	All Cows	RGtreat-SC/PL	RGtreat-SC/SL	RGtreat-OC/SL	RG-SCC	RGtime-100
Mastitis	A	8.041	939	324	2.859	924	3.437
	B	25.474	4.687	3.286	11.974	7.055	11.312
	C	9.288	1.007	641	2.969	2.435	3.540
	Total	42.803	6.633	4.251	17.802	10.414	18.289
Lameness	A	9.155	1.596	1.049	3.385	1.037	3.853
	B	27.643	5.995	4.556	12.214	7.276	12.124
	C	11.243	1.996	2.812	2.538	2.865	4.067
	Total	48.041	9.587	8.417	18.137	11.178	20.044

RGtreat-SC/PL: at least one treatment of the same category in previous lactation, RGtreat-SC/SL: at least one previous treatment of the same category in the same lactation, RGtreat-OC/SL: at least one previous treatment of another category in the same lactation, RG-SCC: high SCC in previous milk recording, RGtime-100: DIM ≤ 100.

**Table A4:** The number of other treatments occurring before the classified treatment for cows in RGtreat-OC/SL. Cows in the group could have multiple previous treatments.

		Other Previous Treatments			
Treatment	Farm	Mastitis	Lameness	Metabolic Disorder	Fertility Disorder
Mastitis	A	-	45	64	21
	B	-	299	668	473
	C	-	109	57	39
Lameness	A	81	-	159	66
	B	396	-	671	529
	C	135	-	105	55
